# Synergistic effect between In_2_O_3_ and ZrO_2_ in the reverse water gas shift reaction†

**DOI:** 10.1039/d4ra01372g

**Published:** 2024-05-08

**Authors:** Jiayu Dong, Hong Wang, Guofeng Zhao, Dong Jiang, Haitao Xu

**Affiliations:** a School of Chemical Engineering, East China University of Science and Technology Shanghai 200237 China xuhaitao@ecust.edu.cn; b School of Chemistry and Molecular Engineering, East China University of Science and Technology Shanghai 200237 China jiangdong@ecust.edu.cn; c Key Laboratory of Functional Molecular Solids, Ministry of Education, College of Chemistry and Materials Science, Anhui Normal University Wuhu 241002 China gfzhao@chem.ecnu.edu.cn; d Institute of Optical Functional Materials for Biomedical Imaging, School of Chemistry and Pharmaceutical Engineering, Shandong First Medical University, Shandong Academy of Medical Sciences Taian 271016 China

## Abstract

Efficient activation of CO_2_ at low temperature was achieved through the interface effect between In_2_O_3_ and ZrO_2_ by their geometric and electronic effects. The results show that 75In_2_O_3_–25ZrO_2_ (In_2_O_3_ : ZrO_2_ molar ratio of 3 : 1), as a catalyst for the reverse water gas shift reaction, can achieve 28% CO_2_ conversion with 96% CO selectivity at 400 °C, 0.1 MPa, a H_2_ : CO_2_ molar ratio of 3 : 1 and a gas hourly space velocity of 10 000 mL g^−1^ h^−1^. *In situ* FTIR experiments provide a basis for clarifying the pivotal role of formate (facilitated at In_2_O_3_–ZrO_2_ interface) in this reaction.

## Abstract 2

The 75In_2_O_3_–25ZrO_2_ forms a solid solution structure, and can achieve 28% CO_2_ conversion and 96% CO selectivity during a 200 h-test.

## Graphical abstract

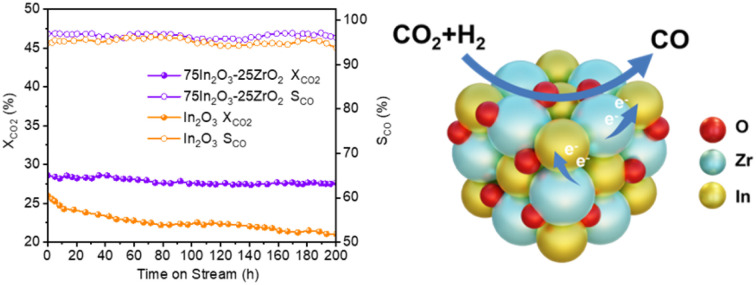

## 1. Introduction

Throughout the course of industrial development, humans have heavily relied on fossil fuels to meet the substantial demand for energy, resulting in a continuous increase in greenhouse gas emissions and exacerbation of climate change.^1^ Utilizing carbon dioxide, an abundant and economical carbon resource, to produce high-value-added chemicals or liquid fuels is of significant importance for energy conservation, emissions reduction, and the sustainable utilization of carbon resources.^2^ In recent years, carbon capture and utilization (CCU) technology has attracted much attention and is considered as one of the useable ways to reduce CO_2_ emissions.^3–7^ The thermal catalytic reduction of CO_2_ refers to the process of converting CO_2_ into hydrocarbons or carbon monoxide (CO) with green hydrogen, typically carried out with the aid of catalysts at elevated temperature.^8^ The rapid development of renewable energy lowers the cost of green hydrogen production,^9^ prompting the urgent need for catalysts with high activity, selectivity, and stability.

The reverse water gas shift (RWGS) reaction hydrogenates CO_2_ into CO, which can be further used to synthesize methanol, breaking through the thermodynamic equilibrium limit of direct methanol production from CO_2_,^10,11^ and can also be combined with Fischer–Tropsch synthesis (FTS) process to prepare useful chemicals such as olefins.^12–15^ Whether producing methanol through the CAMERE method (carbon dioxide hydrogenation to form methanol *via* a RWGS reaction) or preparing low-carbon olefins *via* the CO_2_-FTS method, the RWGS reaction with high CO yield is a crucial step. Therefore, the RWGS reaction is considered as the most promising and prospective pathway in re-utilizing CO_2_.

Catalysts used in the RWGS reaction can be classified into noble metal catalysts, such as Rh,^16^ Ru,^17^ and Pt,^18^ and non-noble metal catalysts, such as Co,^19^ Fe,^20,21^ and Mo.^22,23^ The noble metal catalysts exhibit outstanding performance due to their effective hydrogen dissociation capabilities, but their high costs and instability (nanoparticle agglomeration) limit their industrial application; the non-noble metal catalysts need high temperature to deliver the same performance as noble metal ones.^24^ Therefore, there is of significant importance in developing low-temperature, high-performance catalysts to address these limitations. In recent years, indium oxide (In_2_O_3_) has been found as a proficient catalyst for CO_2_ hydrogenation, with its pronounced catalytic activity attributed to the abundant oxygen vacancy (O_v_) on its surface.^25–28^ Furthermore, In_2_O_3_ can be easily supported and/or modified by promoters to form more O_v_ sites, thereby activating more CO_2_ molecules, and stabilizing surface intermediates near O_v_.^29–33^

Moreover, ZrO_2_ is also used as catalyst support in RWGS reaction, but its role plays in the reaction is still unclear.^34^ Unfortunately, there are relatively few reports related to the synergistic interfacial effect between In_2_O_3_ and ZrO_2_, hampering the rational design of mixed oxides for the RWGS reaction. For the optimal 75In_2_O_3_–25ZrO_2_ (In_2_O_3_ : ZrO_2_ molar ratio of 3 : 1) with abundant In_2_O_3_–ZrO_2_ interface, 28% CO_2_ conversion and 96% CO selectivity can be achieved at 400 °C, 0.1 MPa, H_2_ : CO_2_ molar ratio of 3 : 1 and GHSV (gas hourly space velocity) of 10 000 mL g^−1^ h^−1^. Control experiments and characterization results testify that the as-formed oxygen vacancies (O_vs_) caused by the reduction of In_2_O_3_ to In_2_O_3−*x*_ significantly enhance catalytic activity for 75In_2_O_3_–25ZrO_2_. In addition, *in situ* Fourier transform infrared spectroscopy (FTIR) shows that HCOO* (formate) plays an important role in this reaction. For 75In_2_O_3_–25ZrO_2_ with abundant In_2_O_3_–ZrO_2_ interface, HCOO* is easily hydrogenated into CO. However, for In_2_O_3_, the content of HCOO* is relatively lower, thus contributing to its lower catalytic activity. For ZrO_2_, the CO_3_^2−^ is relatively stable, correlating well with its low catalytic activity. This work elucidates the synergistic effect between mixed In_2_O_3_ and ZrO_2_, paving a way to design industrial catalyst with abundant In_2_O_3_–ZrO_2_ interface to offer excellent catalytic performance for RWGS reaction.

## 2. Experimental

### 2.1. Catalyst preparation

The mixed In–Zr oxides were synthesized by a co-precipitation method. For instance, for the 75In_2_O_3_–25ZrO_2_ (In_2_O_3_ : ZrO_2_ molar ratio of 3 : 1), 1.5612 g In(NO_3_)_3_·*x*H_2_O and 0.3713 g Zr(NO_3_)_4_·5H_2_O were dissolved in 20 mL deionized water, followed by the addition of the mixed solution of NH_4_OH (10 mL, 25 wt% in H_2_O, Alfa Aesar) and ethanol (30 mL, Titan) until the pH reaching 9.2. The resulting slurry was heated to 80 °C with vigorous stirring and aged for 30 min. Then the solid was separated by high-pressure filtration, washed with 500 mL deionized water, dried at 60 °C for 12 h, and calcined at 500 °C (heating rate of *ca.* 2 °C min^−1^) for 3 h. Other catalysts such as In_2_O_3_, ZrO_2_, and *a*In_2_O_3_–*b*ZrO_2_ (*a* and *b* represent In_2_O_3_ and ZrO_2_ molar ratio (*a* = 25%, 50%, and 75%, *b* = 1 − *a*)) were prepared using the same method by simply tuning the molar ratio of In(NO_3_)_3_·*x*H_2_O and Zr(NO_3_)_4_·5H_2_O.

### 2.2. Catalyst characterization

The N_2_ sorption was conducted using the ASAP 2020 instrument (Mack, USA). The specific surface area (*S*_BET_) was determined by Brunauer–Emmett–Teller (BET) model and the pore size was calculated by Barret–Joyner–Halenda (BJH) model. The In and Zr loadings were detected by an inductively coupled plasma-atomic emission spectrometry (ICP-AES) at 167–785 nm/725 instrument (Agilent Corporation, USA). The power X-ray diffraction (XRD) patterns of catalysts were obtained on a Rigaku D/Max 2550 VB/PC instrument (Rigaku, Japan) using a scanning rate of 10° min^−1^. The fine structures were observed by a transmission electron microscopy (TEM) at an accelerating voltage of 200 kV on JEM-2100 (JEOL, Japan). The energy dispersive X-ray spectroscopy (EDX) was measured by the JEM-2100 (JEOL, Japan) with an amplification of 8000–300 000. X-ray photoelectron spectroscopy (XPS) was measured at ESCALAB 250Xi photoelectron spectrometer (Thermo Fisher Scientific, USA) equipped with an Al-Kα X-ray source. All the binding energies were calibrated on the basis of the internal standard of the binding energy of C 1s (284.8 eV). Electron paramagnetic resonance (EPR) spectroscopy was performed using the CIQTEK EPR200-Plus. Spectra were collected accumulating 1 scan for field sweeps of 3250–3850 G at 298 K with a magnetic field modulation frequency of 100 kHz. The spectrum of an empty tube was subtracted to correct for the background signal.

The experiments of H_2_-temperature programmed reduction (H_2_-TPR) and CO_2_-temperature programmed desorption (CO_2_-TPD) were carried out on a ChemBET Pulsar automatic adsorption apparatus (Quantachrome Company, USA) equipped with a thermal conductivity detector (TCD), and the efflux were monitored by an on-line mass spectrometer (MS, SHP8400PMS-L, Shanghai Sunny Hengping Scientific Instrument Co. Ltd, China). For H_2_-TPR, each catalyst (0.1 g) was pretreated in Ar flow (30 mL min^−1^) at 300 °C for 0.5 h and cooled down to room temperature. Then, the gas was switched to H_2_/Ar flow (10 vol% H_2_, 50 mL min^−1^) and the catalyst was reduced from room temperature to 800 °C at a heating rate of 10 °C min^−1^. For CO_2_-TPD, each catalyst (0.1 g) was pretreated in Ar flow (30 mL min^−1^) at 400 °C for 1 h, and then reacted in mixture gas (the molar ratio of H_2_ : CO_2_ is 3 : 1, 50 mL min^−1^) at 400 °C for 2 h. Then, the catalyst was cooled to 50 °C in the same flow followed by CO_2_ (50 mL min^−1^) adsorption at 50 °C for 2 h. After that, the catalyst was flushed in He flow (50 mL min^−1^) for 0.5 h, followed by heated from 50 to 800 °C at a rate of 10 °C min^−1^, and signals of CO_2_ were monitored by MS on line.

The *in situ* Fourier transform infrared (FT-IR) was conducted on a IRPrestige-21 equipment (Shimadzu, Japan). A resolution of 8 cm^−1^ and scanning times of 50. 50 mg catalyst and 100 mg KBr were pressed into a wafer and placed in the *in situ* chamber. All the samples were pretreated at 400 °C in H_2_ flow (37.5 mL min^−1^) for 10 min and cooled to the room temperature to obtain the background spectrum. When the adsorption of CO_2_, the flow was switched to CO_2_ (12.5 mL min^−1^, 99.99%) at room temperature for 10 min, after that, CO_2_ was switched off and the catalyst was maintained at 50 °C for 2 h. Subsequently, catalyst was purged with a He flow (30 mL min^−1^) for 5 minutes and then raised from 50 to 400 °C, with the spectra were collected. After raising to 400 °C, the flow was switched to H_2_ for 10 s, H_2_ was switched off and the spectra was collected at 0.5 MPa. When the co-adsorption of CO_2_ and H_2_, the flow was switched to the mixed gas (the molar ratio of H_2_ : CO_2_ is 3 : 1, 50 mL min^−1^). The temperature was raised from 100 to 400 °C and the spectra were collected.

### 2.3. Catalytic evaluation

In this work, a continuous fixed-bed reactor was used to evaluate the performance of catalysts. Typically, 0.3 g catalyst was loaded into a reactor with an inner diameter of 7 mm and the length of 700 mm. H_2_ (36 mL min^−1^), CO_2_ (12 mL min^−1^), and Ar (2 mL min^−1^) was controlled by mass flow controllers, forming a H_2_/CO_2_/Ar (molar ratio of 72/24/4) mixture and passing through the catalyst bed. The Ar was used as the internal standard gas. Then, the temperature was successively raised from room temperature to 400 °C and maintained for 2 h. The effluent was analyzed by online gas chromatography (GC7900), equipped with a thermal conductivity detector (TCD) and TDX-1 column. The CO_2_ conversion (*X*_CO_2__), CO selectivity (S_CO_), CO yield (Y_CO_) and CH_4_ selectivity (*S*_CH_4__) were calculated as follows:1
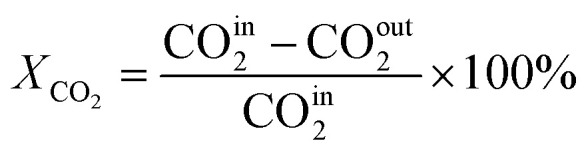
2
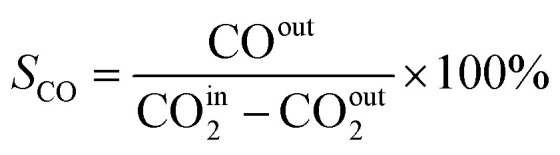

3 *Y*_CO_ = *X*_CO_2__ × *S*_CO_

4
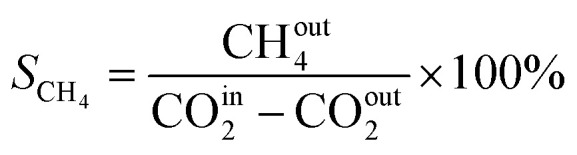
CO^in^_2_ and CO^out^_2_ represent the concentration of CO_2_ at the inlet and outlet, respectively; CO^out^ represents the concentration of CO at the outlet; CH^out^_4_ represent the concentration of CO at the outlet.

## 3. Results and discussion

### 3.1. Structures and chemical states of fresh catalysts

Five In_2_O_3_–ZrO_2_ catalysts were prepared by the co-precipitation method, varying the molar content of In_2_O_3_ of 0, 25%, 50%, 75% and 100%. The inductively coupled plasma-atomic emission spectrometry (ICP-OES) measurements confirmed that In and Zr contents were almost identical to the theoretical value. Adding ZrO_2_ to In_2_O_3_ will slightly increase the specific surface area (Table S1†), but the specific surface area of 75In_2_O_3_–25ZrO_2_ is close to that of In_2_O_3_, albeit the catalyst area is not the key factor determining catalytic activity.^35^ Moreover, the type IV hysteresis loop testifies the mesoporous structure of this series of catalysts (Fig. S1†). TEM images of this series of catalysts show the similar morphologies, with the diameter of 8–15 nm (Fig. S2†).

For 75In_2_O_3_–25ZrO_2_, the average particle size is 10.0 ± 1.4 nm, and the HRTEM images illustrate the lattice distances of 0.292, 0.275, and 0.297 nm, corresponding to the In_2_O_3_(222), In_2_O_3_(321), and t-ZrO_2_(101) planes, respectively (Fig. S3a and b†). The STEM-EDX mapping images show that In and Zr elements are randomly distributed on the catalyst surface (Fig. S3c and d†), forming abundant In_2_O_3_–ZrO_2_ interface and tentatively contributing to excellent catalytic performance.

X-ray diffraction (XRD) was used to figure out the effect of Zr modification on bulk structure. The XRD patterns in Fig. S4a.† display that the pure ZrO_2_ (*i.e.*, 0In_2_O_3_–100ZrO_2_) prefers to crystallize to its thermodynamically stable monoclinic structure; the presence of In steers the growth of ZrO_2_ toward metastable tetragonal phase,^28^ suggesting that partial In is incorporated into the ZrO_2_ lattice in the form of In–O–Zr bond, as evidenced by the HRTEM image of 75In_2_O_3_–25ZrO_2_ sample.^36^ The transition from In–O–In bond to In–O–Zr bond should greatly improve the CO_2_ conversion and CO selectivity of In_2_O_3_–ZrO_2_ catalysts (see the results in Section 3.2). Owing to the fact that the lattice parameters of cubic In_2_O_3_ (JCPDS card 06-0416) and t-ZrO_2_ (JCPDS card 37-1413) are akin, their XRD patterns are virtually identical. However, as shown in Fig. S4b,† the diffraction peak moves from 30.167° (t-ZrO_2_(111)) to 30.580° (c-In_2_O_3_(222)) with the increase of In_2_O_3_ content, and such tiny peak shift confirms the generation of In_2_O_3_–ZrO_2_ solid solution.

**Fig. 1:**
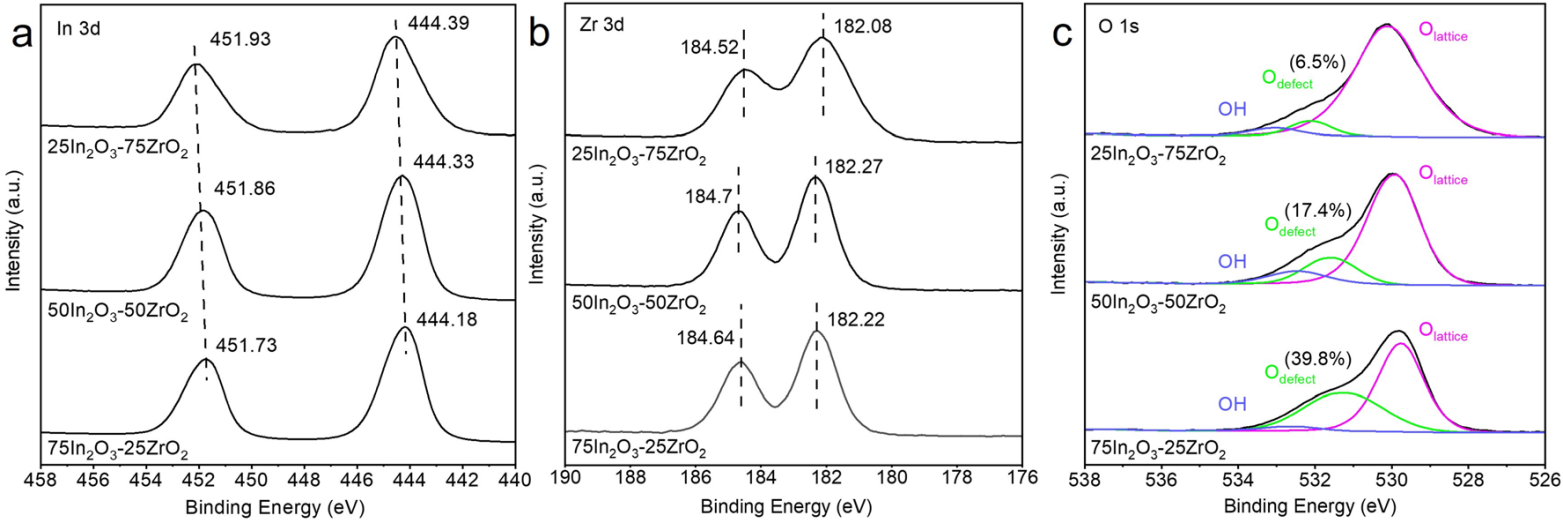
XPS spectra of (a) In 3d, (b) Zr 3d, and (c) O 1s for the 25In_2_O_3_–75ZrO_2_, 50In_2_O_3_–50ZrO_2_ and 75In_2_O_3_–25ZrO_2_ catalyst.

The surface chemical states of In_2_O_3_–ZrO_2_ catalysts were characterized by XPS (Fig. 1) and EPR (Fig. S6†). The symmetric binding energy peaks at ∼452 and ∼444.3 eV testify that In species exists in the form of In^3+^.^37^ With the increase of In_2_O_3_ content, the binding energy of In^3+^ decreases slightly, indicating the electron transfer from Zr to In.^36^ The symmetric binding energy peaks at ∼184.5 and ∼182.0 eV testify that Zr species exists in the form of Zr^4+^.^38^ For 50In_2_O_3_–50ZrO_2_ and 75In_2_O_3_–25ZrO_2_, the binding energies of Zr are higher that of 25In_2_O_3_–75ZrO_2_, also coinciding with the electron transfer. For the O 1s XPS spectra, the major peak at 529.5–531.0 eV corresponds to lattice oxygen, the peak at 531.0–532.0 eV to O_v_, and the one at 532.5–533.0 eV to surface OH.^39^ Obviously, with the increase of In_2_O_3_ content, the O_v_ content increases progressively. Fig. S5† shows that there is a positive correlation between the CO STY (space-time yield) and the oxygen vacancy concentration, which means that the O_v_ may play an important role in the RWGS reaction. Furthermore, the EPR results in Fig. S6† reveals a signal of *g* = 1.890 for fresh In_2_O_3_, which implies that the surface vacancies exist on In_2_O_3_.^40^ Pure ZrO_2_ sample exhibits an isotropic EPR signal at *g* = 1.973, which is assigned to the bulk Zr^3+^ ions located at axially symmetric sites. The 75In_2_O_3_–25ZrO_2_ demonstrates a prominent signal that can be attributed to unpaired electrons trapped in symmetric site at *g* = 2.004, which is always typically assigned to oxygen vacancies.^41^ This means the synergistic effect between In_2_O_3_ and ZrO_2_ in 75In_2_O_3_–25ZrO_2_ solid solution is beneficial to produce new oxygen vacancies at *g* = 2.004, which is in line with the XPS result.

H_2_ temperature-programmed reduction (H_2_-TPR) tests were conducted to determine the reactivity of the In_2_O_3_–ZrO_2_ catalyst toward H_2_ activation in the temperature range of 50–800 °C, as shown in Fig. S7a.† The H_2_-TPR profiles revealed that reduction temperature of bulk In_2_O_3_ in In_2_O_3_ and 75In_2_O_3_–25ZrO_2_ are 662 °C and 697 °C respectively, while the reduction temperature of surface In_2_O_3_ are 189 °C and 225 °C respectively. However, the H_2_-TPR of ZrO_2_ demonstrates no significant H_2_ consumption, which means the neglectable reducibility of ZrO_2_. Interestingly, for 75In_2_O_3_–25ZrO_2_, the reduction signals of surface and the bulk In_2_O_3_ are located at a higher temperature than that of pure In_2_O_3_ catalyst, hinting a stronger interaction between In_2_O_3_ and ZrO_2_.^29^ This also shows the increasing O_v_ content over 75In_2_O_3_–25ZrO_2_ catalyst, which is in accordance with the XPS result and the prominent catalytic activity.^42^

CO_2_ temperature programmed desorption (CO_2_-TPD) was conducted to further investigate the CO_2_ adsorption behaviour on the In_2_O_3_–ZrO_2_ catalyst, as shown in Fig. S7b.† The profiles exhibit several significant CO_2_ evolution signals from the ZrO_2_ and 75In_2_O_3_–25ZrO_2_ catalyst in the temperature range of 134–220, 273–315 and 396–477 °C. While the signal of CO_2_ adsorbed on pure In_2_O_3_ are not detectable. The signal peak around 153 °C belongs to the physisorption of CO_2_. Other signal peaks belong to the chemically absorbed CO_2_ on the H_2_-induced oxygen vacancy sites (O_v_).^42^ Additionally, CO_2_-TPD has been widely used to measure the surface basicity of catalysts, and high desorption temperature promised a strong basic site.^43^ Compared with ZrO_2_ catalyst, the CO_2_ desorption peak of 75In_2_O_3_–25ZrO_2_ catalyst shift to the higher temperatures of 315 °C and 75In_2_O_3_–25ZrO_2_ catalyst have strong site at around 450 °C. Specifically, the addition of In enhances the strength of CO_2_ adsorption on these sites, owing to the increase in basic intensity.^42^ The characterization results of H_2_-TPR and CO_2_-TPD consistently confirm that In_2_O_3_–ZrO_2_ interface benefits the formation of oxygen vacancies, thus enhancing the ability of 75In_2_O_3_–25ZrO_2_ catalyst to CO_2_ adsorption and H_2_ activation.

### 3.2. Catalytic performance

CO_2_ hydrogenation mainly involves the following three reactions (5)–(7) to produce three products of CO, CH_4_ and CH_3_OH, respectively.

5 CO_2_ + H_2_ ↔ CO + H_2_O, *Δ*_r_*H*^θ^_m_ = 41.2 kJ mol^−1^

6 CO_2_ + 4H_2_ ↔ CH_4_ + H_2_O, *Δ*_r_*H*^θ^_m_ = −164.9 kJ mol^−1^

7 CO_2_ + 3H_2_ ↔ CH_3_OH + H_2_O, *Δ*_r_*H*^θ^_m_ = −49.4 kJ mol^−1^

**Fig. 2:**
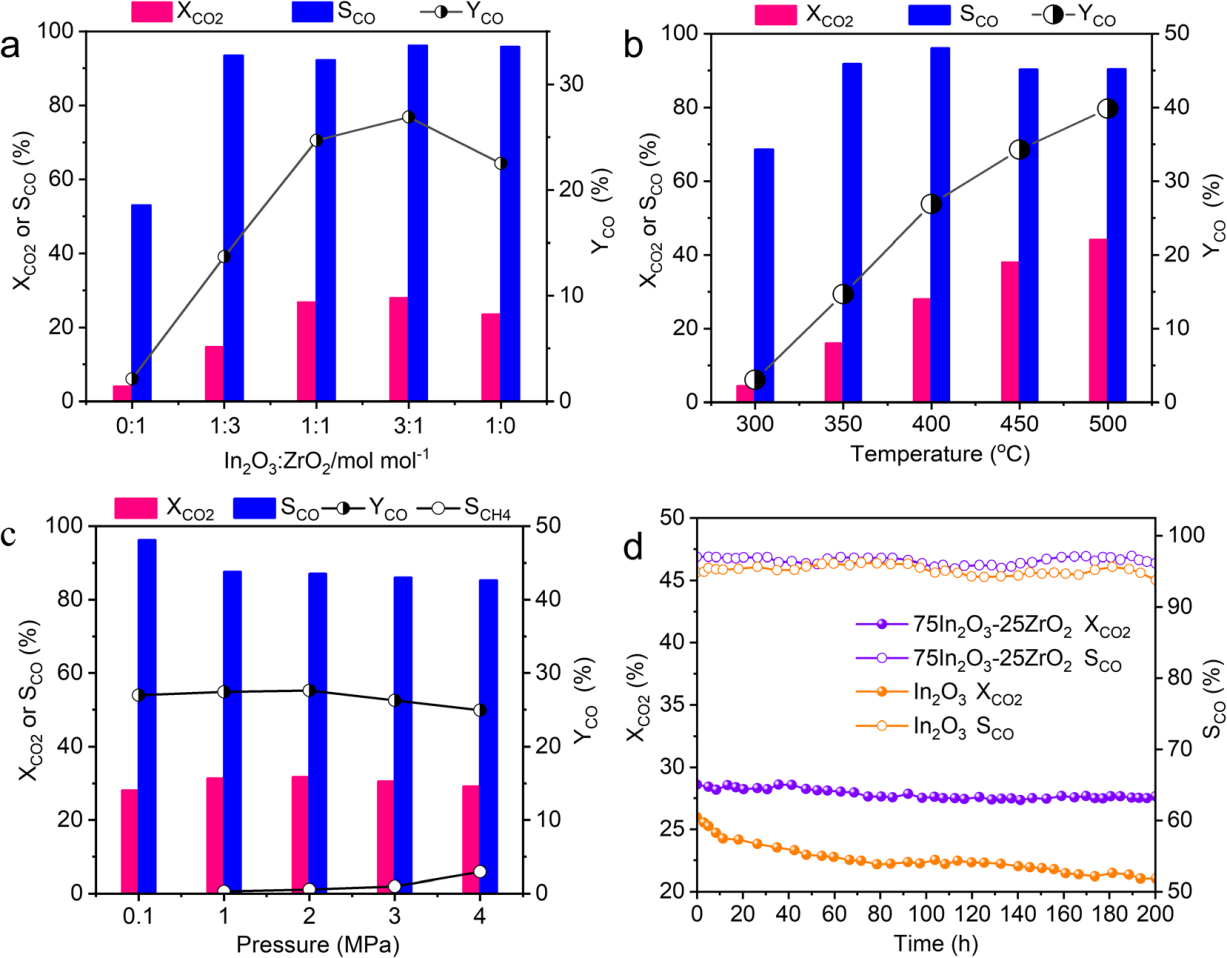
(a) The catalytic performance of the In_2_O_3_–ZrO_2_ catalysts with different In_2_O_3_ ratio (reaction conditions: 400 °C, 0.1 MPa, H_2_ : CO_2_ : Ar ratio = 72 : 24 : 4, 10 000 mL g^−1^ h^−1^); (b) influence of reaction temperature on the catalytic performance of 75In_2_O_3_–25ZrO_2_ (reaction conditions: 0.1 MPa, H_2_ : CO_2_ : Ar ratio = 72 : 24 : 4, 10 000 mL g^−1^ h^−1^); (c) influence of pressure on the catalytic performance of 75In_2_O_3_–25ZrO_2_ (reaction conditions: 400 °C, H_2_ : CO_2_ : Ar ratio = 72 : 24 : 4, 10 000 mL g^−1^ h^−1^); (d) the stability test of 75In_2_O_3_–25ZrO_2_ and In_2_O_3_ (reaction conditions: 400 °C, 0.1 MPa, H_2_ : CO_2_ : Ar ratio = 72 : 24 : 4, 10 000 mL g^−1^ h^−1^).

Fig. 2a shows the CO_2_ conversion, CO selectivity, and CO yield over the five catalysts. The catalytic performance of pure ZrO_2_ (*i.e.*, 0In_2_O_3_–100ZrO_2_) is extremely poor, with CO_2_ conversion of only 4% and CO selectivity of only 53%, while the pure In_2_O_3_ (*i.e.*, 100In_2_O_3_–0ZrO_2_) gives higher CO_2_ conversion of 23.5% and CO selectivity of 95.8%. Interestingly, the In_2_O_3_–ZrO_2_ catalysts (*i.e.*, 25In_2_O_3_–75ZrO_2_, 50In_2_O_3_–50ZrO_2_, and 75In_2_O_3_–25ZrO_2_) all offers CO selectivity above 92%, with volcano evolution of CO_2_ conversion. Most notably, the 75In_2_O_3_–25ZrO_2_ offers the highest CO selectivity of 96% and highest CO_2_ conversion of 28%. Due to a pronounced synergistic effect between ZrO_2_ and In_2_O_3_, the In–Zr interface within the bimetallic oxides augments the density of O_v_ on the In_2_O_3_ surface, thereby significantly enhancing the adsorption and hydrogenation capacities towards CO_2_. In addition, no methane can be detected, and a small amount of methanol was the only by-product.

The influence of reaction temperature, pressure, and gas hourly space velocity (GHSV) on catalytic performance is exhibited in Fig. 2b, c and S8.† At 0.1 MPa, and GHSV of 10 000 mL g^−1^ h^−1^, with the temperature rising from 300 to 500 °C, CO_2_ conversion increases from 4% to 44%, and the highest CO selectivity is 96% at 400 °C. At 400 °C, and GHSV of 10 000 mL g^−1^ h^−1^, with the pressure increasing from 0.1 to 4 MPa, CO_2_ conversion slightly increases from 28% to 29%, but CO selectivity decreases from 96% to 85% (with the formation of new by-product CH_4_), because high reaction pressure is beneficial to CO_2_ methanation reaction.^44^ Moreover, at 0.1 MPa, and 400 °C, CO_2_ conversion decreases from 35% to 27.7% with increasing GSHV from 6000 to 14 000 mL g^−1^ h^−1^, while the maximum CO selectivity is 94% at the GSHV of 10 000 mL g^−1^ h^−1^. Hence, the optimized reaction condition is as follows: 0.1 MPa, 400 °C and GHSV of 10 000 mL g^−1^ h^−1^. For the best catalyst 75In_2_O_3_–25ZrO_2_, under the best reaction conditions, the CO_2_ conversion and CO selectivity are 28% and 96% in the 200 h-test. However, for In_2_O_3_, the conversion decreases from 26% to 21%. Compared with pure In_2_O_3_, the stability of mixed oxides is obviously enhanced. Hence, the In_2_O_3_–ZrO_2_ interface is of great importance in improving and maintaining catalytic activity (Fig. 2d). We compared the catalyst 75In_2_O_3_–25ZrO_2_ with other catalysts including non-noble metal and noble metal catalysts in the RWGS reaction in Table S3.† CO_2_ conversion, CO selectivity and STY of 75In_2_O_3_–25ZrO_2_ are very promising. Notably, the STY of 75In_2_O_3_–25ZrO_2_ is higher than other catalysts (apart from Ag/Al_2_O_3_). Furthermore, compared with noble metal catalysts, In-based catalysts have lower cost and more prospects in industry applications.

### 3.3. Surface intermediates and reaction mechanism

**Fig. 3:**
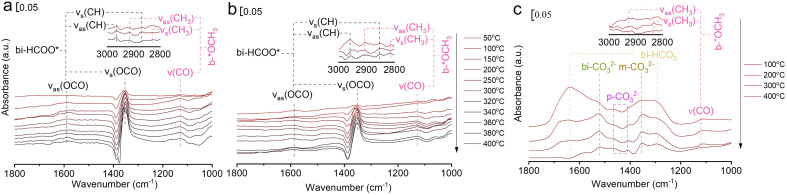
*In situ* FTIR spectra of CO_2_ adsorption at different temperatures over (a) 75In_2_O_3_–25ZrO_2_, (b) In_2_O_3_, and (c) ZrO_2_.

*In situ* FTIR was used to investigate the evolution of key surface intermediates for RWGS reaction, and the wavenumbers of the intermediates are summarized in Table S4.†^33,34,36,37,39,45–55^ Firstly, the three catalysts (75In_2_O_3_–25ZrO_2_, In_2_O_3_, and ZrO_2_) were placed into the chamber and reduced with hydrogen at 400 °C. Subsequently, CO_2_ was introduced into the chamber for adsorption. Finally, the gaseous CO_2_ was purged by He flow and the spectra were collected from 50 to 400 °C (Fig. 3). For 75In_2_O_3_–25ZrO_2_ (Fig. 3a), the following characteristic bands can be observed: bi-HCOO* (bidentate formate, at 1350, 1589, 2873 and 2967 cm^−1^);^33,36,39,46,48,50,52–55^ b-*OCH_3_ (bridged methoxy, at 1128, 2822 and 2930 cm^−1^).^33,39,50–53^ With the increase of temperature, the peak area of bi-HCOO* increases significantly, testifying that CO_2_ could be transformed into bi-HCOO* easily. For In_2_O_3_ (Fig. 3b), similar characteristic bands are also found, but the content of bi-HCOO* is relatively lower, corresponding well with its lower catalytic activity and tentatively showing that bi-HCOO* may play an important role in this reaction. For ZrO_2_ (Fig. 3c), the following characteristic bands can be observed: bi-HCO_3_^−^ (bidentate bicarbonate, at 1284 and 1636 cm^−1^);^34,49,54^ m-CO_3_^2−^ (monodentate carbonate, at 1355 cm^−1^);^34,49^ bi-CO_3_^2−^ (bidentate carbonate, at 1523 cm^−1^);^34,47^ p-CO_3_^2−^ (polydentate carbonate, at 1463 and 1411 cm^−1^);^47,48,54^ b-*OCH_3_ (bridged methoxy, at 1126, 2830 and 2925 cm^−1^).^33,39,50–53^ With the increase of temperature, bi-HCO_3_^−^ decomposes rapidly, while bi-CO_3_^2−^ and m-CO_3_^2−^ decompose sluggishly. Because of with strong thermal resistance and a rather low separation between the two C–O stretching modes, polydentate carbonate species is relatively stable (*ν*_as_(CO_3_) = 1463 cm^−1^ and *ν*_s_(CO_3_) = 1411 cm^−1^).^54^ In addition, the peak area of p-CO_3_^2−^ increases slightly, indicating that the above species may transform into p-CO_3_^2−^.^37^

**Fig. 4:**
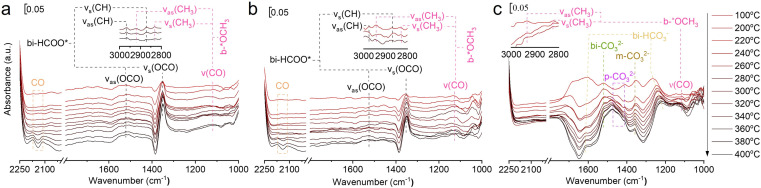
*In situ* FTIR spectra of the reaction of CO_2_ and H_2_ over (a) 75In_2_O_3_–25ZrO_2_, (b) In_2_O_3_, and (c) ZrO_2_.

In order to testify the pivotal role of bi-HCOO* playing in this reaction, the reaction of H_2_ and CO_2_ (molar ratio of H_2_ and CO_2_ is 3 : 1) over these three reduced catalysts were tracked by *in situ* FTIR (Fig. 4). For 75In_2_O_3_–25ZrO_2_, the characteristic band of CO is observed at 320 °C (*ν*(CO) = 2111.1 and 2170 cm^−1^). However, for In_2_O_3_, CO starts to appear at 360 °C, corresponding well with its lower catalytic activity. For ZrO_2_, the characteristic bands of CO are not observed, showing that bi-HCO_3_^−^, bi-CO_3_^2−^, m-CO_3_^2−^, and p-CO_3_^2−^ can't be hydrogenated easily.^48^ Lastly, for all the three catalysts, 
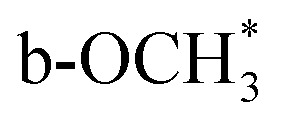
 is also observed, but this species could only be hydrogenated to CH_4_ at relative higher 0.5 MPa, thus excluding the role of 
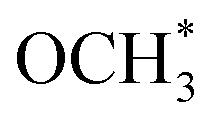
 playing under the reaction conditions (Fig. S9†). But the CH_4_ is not formed in the real fixed-bed reaction process, which is likely caused by the different conditions between *in situ* FTIR and real reaction process.

Combined with the above analyses, it can be suggested that CO_2_ hydrogenation on the In_2_O_3_–ZrO_2_ catalyst through HCOO* intermediates (Scheme S1†). H_2_ adsorbed on the exposed surface of In_2_O_3_ crystal to form 
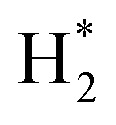
, and then formed 
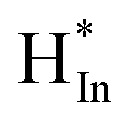
 and 
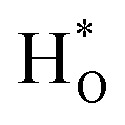
 at In site and O site, respectively. At the same time, CO_2_ is adsorbed on a base on the surface of the composite oxide, activated by oxygen vacancy, and then combined with activated 
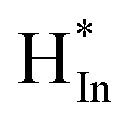
 to form formate intermediate (HCOO*). HCOO* interacts with the site of ZrO_2_, undergoes the cleavage of C–O and C–H bonds, and forms O–H bonds at the same time, producing CO* and OH*, and CO* desorbs to produce CO.^33,37^ In this case, ZrO_2_ can not only modify In_2_O_3_, but also serve as an active site. In_2_O_3_–ZrO_2_ constitutes a bimetallic In–Zr oxide catalyst system.

## 4. Conclusions

In this work, the optimal 75In_2_O_3_–25ZrO_2_ and the contrastive In_2_O_3_, ZrO_2_ were prepared by the coprecipitation method, and 75In_2_O_3_–25ZrO_2_ exhibits excellent 28% conversion and 96% selectivity in the RWGS reaction under the best reaction conditions (400 °C, 0.1 MPa, H_2_ : CO_2_ molar ratio of 3 : 1 and gas hourly space velocity of 10 000 mL g^−1^ h^−1^). XRD and STEM-EDX show that the In_2_O_3_–ZrO_2_ solid solution is formed, and XPS testifies that the electron transfer effect plays an important role in this reaction. *In situ* FTIR shows that: for 75In_2_O_3_–25ZrO_2_ with abundant In_2_O_3_–ZrO_2_ interface, HCOO* is easily hydrogenated into CO; however, for In_2_O_3_, the content of HCOO* is relatively lower, thus contributing to its lower catalytic activity; for ZrO_2_, the CO_3_^2−^ is relatively stable, correlating well with its low catalytic activity. This work definitely testifies the pivotal role of HCOO* in the RWGS reaction, but also paves a way to design bimetal oxide catalyst with excellent catalytic performance for RWGS reaction.

## Conflicts of interest

The authors declare that there is no conflict of interest.

## Supplementary Material

RA-014-D4RA01372G-s001
