# Supplementary material for: Synergistic effect between In2O3 and ZrO2 in the reverse water gas shift reaction
Source: RSC Adv. 2024 May 8;14(21):14868–74. doi: 10.1039/d4ra01372g (PMC11077989; doi:10.1039/d4ra01372g)
Supplement: RA-014-D4RA01372G-s001 [file RA-014-D4RA01372G-s001.pdf]

Electronic Supplementary Material (ESI) for RSC Advances.
This journal is © The Royal Society of Chemistry 2024

Supplementary Information

Synergistic effect between In_2O_3 and ZrO_2 in reverse water gas shift reaction

Jiayu Dong[a], Hong Wang[c], Guofeng Zhao[b,*], Dong Jiang[a,*], Haitao Xu[a,*]

[a]School of Chemical Engineering, East China University of Science and Technology, Shanghai 200237, China

[a]School of Chemistry and Molecular Engineering，East China University of Science and Technology, Shanghai 200237, China

[b]Key Laboratory of Functional Molecular Solids, Ministry of Education, College of Chemistry and Materials Science, Anhui Normal University, Wuhu 241002, China

[c]Institute of Optical Functional Materials for Biomedical Imaging, School of Chemistry and Pharmaceutical Engineering, Shandong First Medical University & Shandong Academy of Medical Sciences, Taian 271016, China

*Corresponding authors. E-mail addresses: gfzhao@chem.ecnu.edu.cn (G. Zhao); jiangdong@ecust.edu.cn (D. Jiang); xuhaitao@ecust.edu.cn (H. Xu)

Table S1 Metal contents and textural properties of In_2O_3-ZrO_2 catalysts

Catalyst	In_2O_3 content[a]	ZrO_2 content[a]	Porous properties[b]	
	Measured (mol %)	Measured (mol %)	S_{BET} ($m^2 \cdot g^{-1}$)	D_{pore} (nm)
100In_2O_3-0ZrO_2	100	0	55	38
75In_2O_3-25ZrO_2	73.6	26.4	60	25
50In_2O_3-50ZrO_2	47.6	52.4	71	15
25In_2O_3-75ZrO_2	23.9	76.2	68	7
0In_2O_3-100ZrO_2	0	100	55	10

[a]Determined by ICP.

[b]Measured by N_2 desorption.

Table S2 Particle sizes and O_v contents of In_2O_3-ZrO_2 catalysts

Catalyst	D (nm)[a]	O_v content[b]
$100In_2O_3$-$0ZrO_2$	15	-
$75In_2O_3$-$25ZrO_2$	11	39.8%
$50In_2O_3$-$50ZrO_2$	10	17.4%
$25In_2O_3$-$75ZrO_2$	11	6.5%
$0In_2O_3$-$100ZrO_2$	11	-

[a]Calculated by In_2O_3(222) or ZrO_2(111) peaks using Scherrer equation.
[b]Measured by XPS.

Table S3 Comparison of catalysts used in RWGS reaction

Catalyst	T(°C)	P(MPa)	H_2/CO_2	X_{CO2}(%)	S_{CO}(%)	STY	Ref
In_2O_3-CeO_2	400	0.1	1:1	5.34	100	0.77	1
Cu5In5/CeO_2	400	0.1	4:1	20	100	0.54	2
Cu5In5/ZrO_2	400	0.1	4:1	<5	100	<0.14	2
Ni/ZrO_2-80	400	0.1	4:1	<10	100	0.41	3
Cs-Fe_xC-co	400	0.1	4:1	36	59	0.76	4
10%Cu-5%Fe/CeO_2	450	0.1	1:1	25	100	0.18	5
Cu-Ni/γ-Al_2O_3	500	0.1	1:1	23.2	75.5	0.11	6
Au/ZrO_2	350	0.3	3:1	~6	100	0.09	7
Au/Al_2O_3	350	0.1	5:1	20	>99	0.24	8
Ag/Al_2O_3	600	0.1	3:1	14	100	0.8	9
75In_2O_3-25ZrO_2	400	0.1	3:1	28	96	0.81	this work

Table S4 Assignment of absorbance peaks observed in the *in-situ* FTIR

Surface species		Vibration mode	Frequency (cm^{-1}) Wavenumber (cm^{-1})	Ref
Bidentate formate	bi-$HCOO^*$	$\nu_{as}(CH)$	2969	10
			2965	11
			2969	12
		$\nu_s(CH)$	2869	10
			2872	11
			2876	12
		$\nu_{as}(OCO)$	1561-1581	11, 13-18
			1583	19
			1585	10
			1588	12
		$\nu_s(OCO)$	1346	11
			1356-1359	16, 18
Monodentate formate	m-$HCOO^*$	$\nu_s(OCO)$	1255	20
			1285	12
			1295	19
			1298	14
Bridged methoxy	b-$^*OCH_3$	$\nu_{as}(CH_3)$	2931	10
			2926	16
			2928	12
		$\nu_s(CH_3)$	2836	10
			2820	16
			2825	12
		$\nu(CO)$	1140-1150	12, 18-20
Bidentate bicarbonate	bi-HCO_3	$\nu_{as}(CO_3)$	1620	21
			1630	14
			1636-1650	22
		$\delta(OH)$	1225	14, 21
			1225-1236	22
Monodentate carbonate	m-CO_3^{2-}	$\nu_{as}(CO_3)$	1355	21
			1368	22
Bidentate carbonate	bi-CO_3^{2-}	$\nu_{as}(CO_3)$	1555	21
			1563	23
Polydentate carbonate	p-CO_3^{2-}	$\nu_{as}(CO_3)$	1460	14
			1474	13
		$\nu_s(CO_3)$	1405	23
			1406	14
Carbon monoxide	CO		2077, 2130	23
			2115	10
			2129	19
Methane	CH_4		3016	24, 25

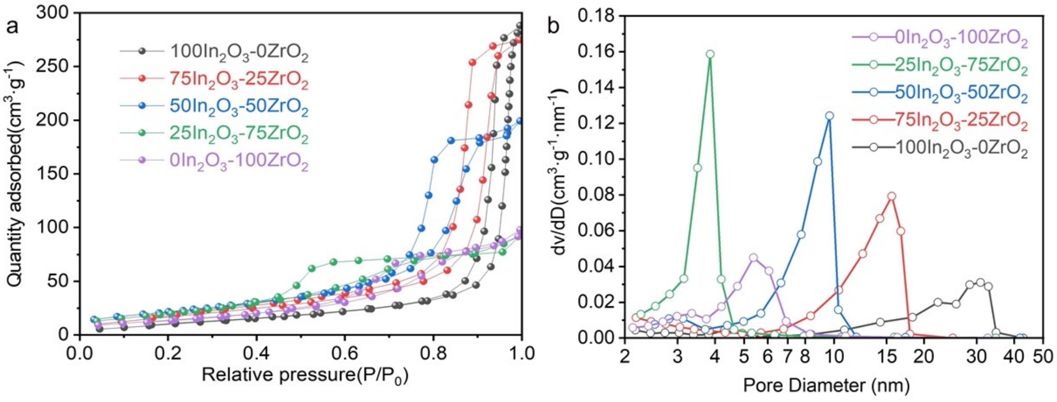

Figure S1 (a) N_2 adsorption-desorption isothermal curves and (b) pore size distribution of In_2O_3-ZrO_2 catalysts.

The N_2 adsorption and desorption curves of all catalysts belong to IV adsorption isotherms, which indicates the mesoporous structure (Fig. S1a). Fig. S1b. shows the pore diameter distribution of the catalysts. As can be observed, all catalysts exhibit maxima in the mesoporous region.

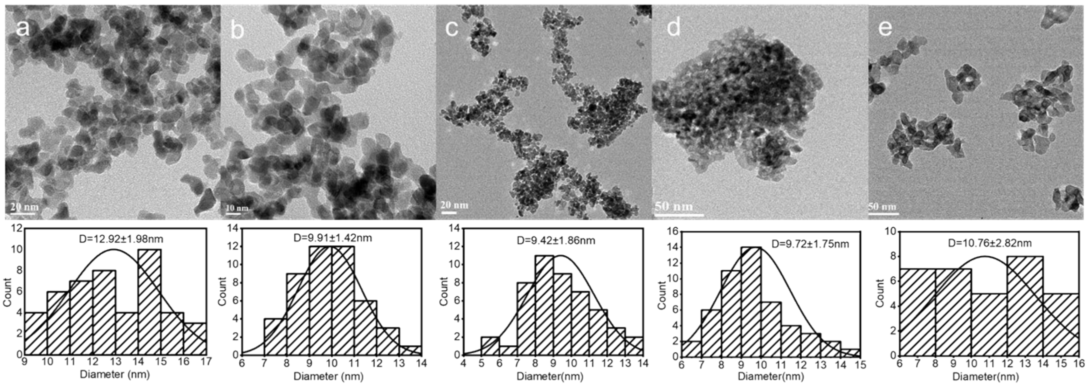

Figure S2 TEM images and particle size distributions of (a) $100In_2O_3$-$0ZrO_2$, (b) $75In_2O_3$-$25ZrO_2$, (c) $50In_2O_3$-$50ZrO_2$, (d) $25In_2O_3$-$75ZrO_2$, and (e) $0In_2O_3$-$100ZrO_2$ catalysts.

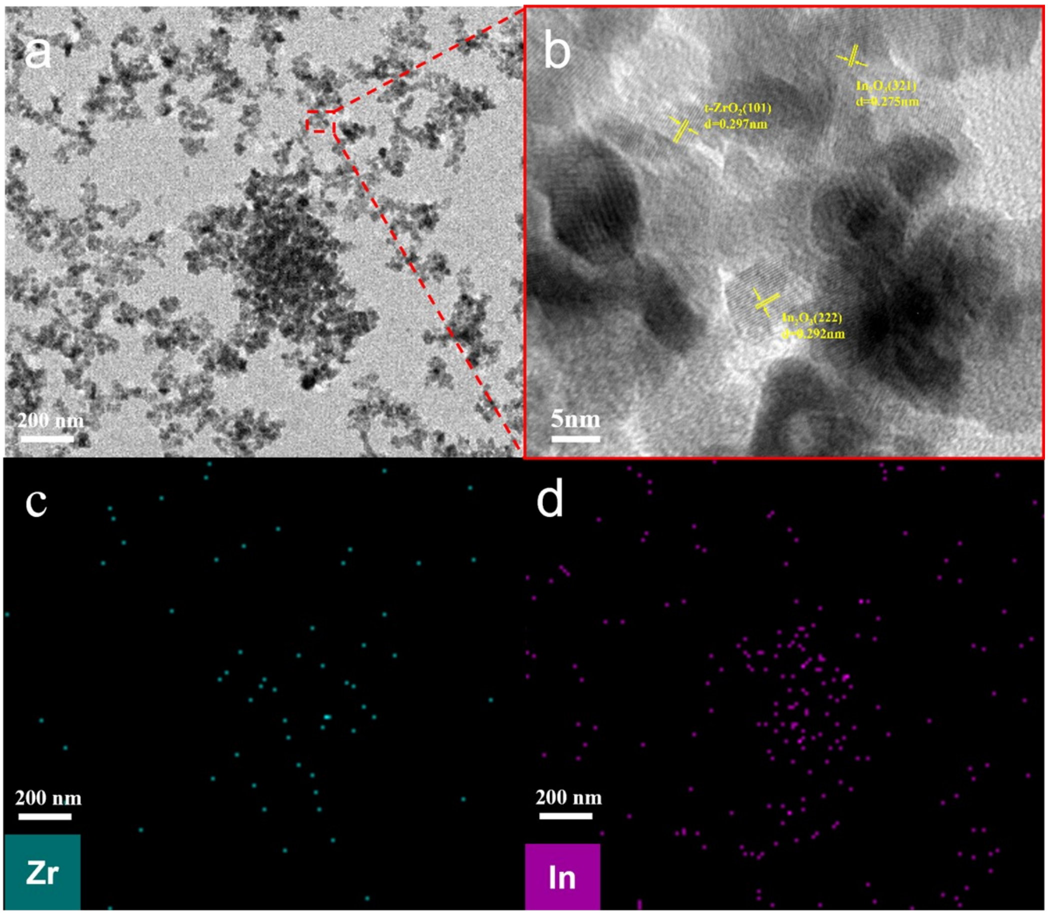

Figure S3 (a) HAADF-STEM image, (b) high-resolution TEM image and (c, d) corresponding energy-dispersive X-ray (EDX) elemental mappings of $75In_2O_3$-$25ZrO_2$ catalyst.

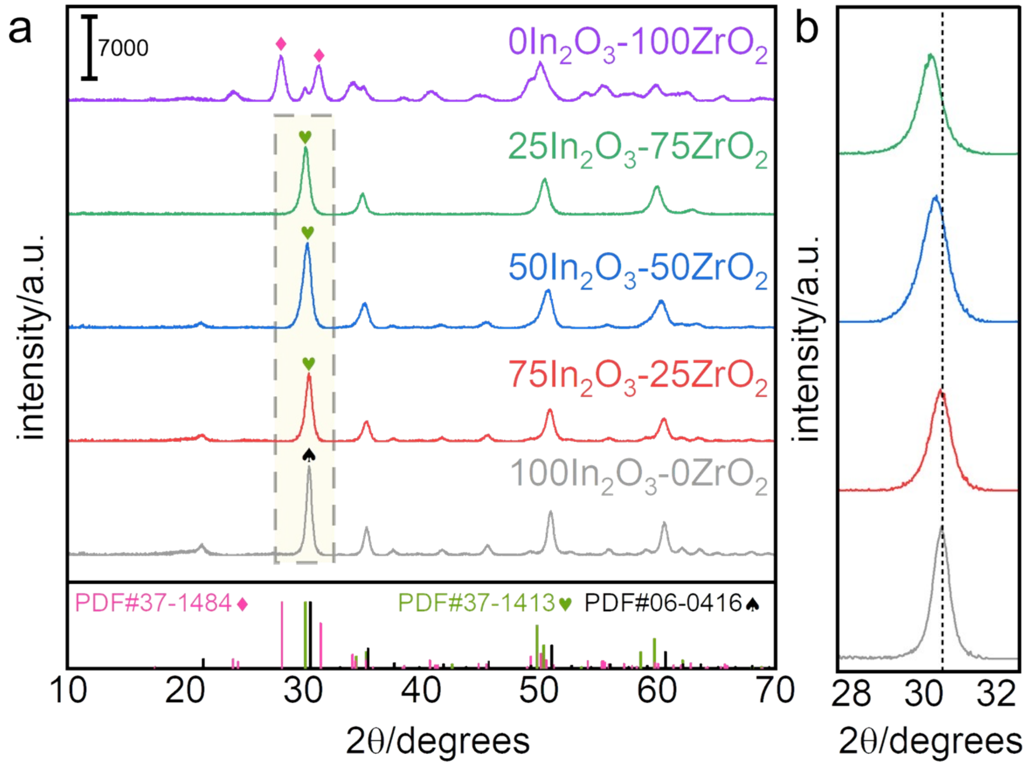

Figure S4 (a) XRD patterns and (b) enlarged parts (28-32°) of In_2O_3-ZrO_2 catalysts.

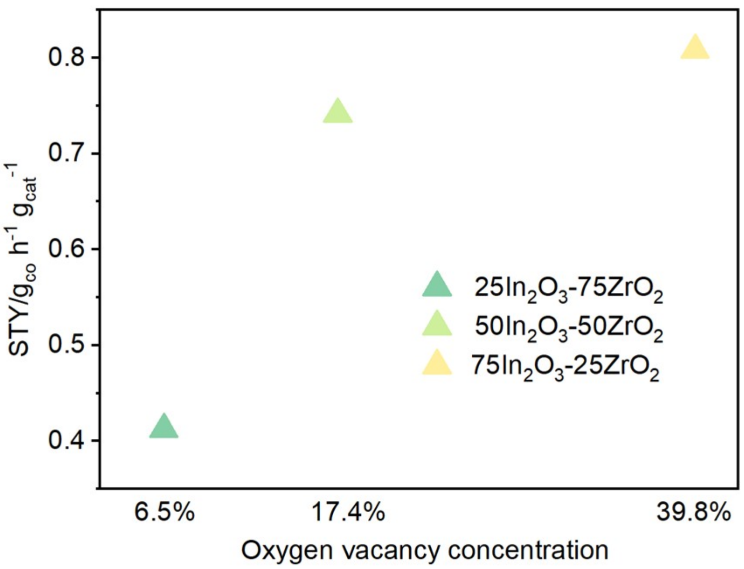

Figure S5 The relationship diagram of CO STY and oxygen vacancy concentration of In_2O_3-ZrO_2 catalysts.

As shown in Fig. S5, the oxygen vacancy concentration of 25In_2O_3-75ZrO_2, 50In_2O_3-50ZrO_2 and 75In_2O_3-25ZrO2 is 6.5%, 17.4% and 39.8%, respectively. And the corresponding STY (space-time yield) is 0.4, 0.7 and 0.8, respectively. The STY of the In_2O_3-ZrO_2 catalyst has a positive correlation with the oxygen vacancy concentration.

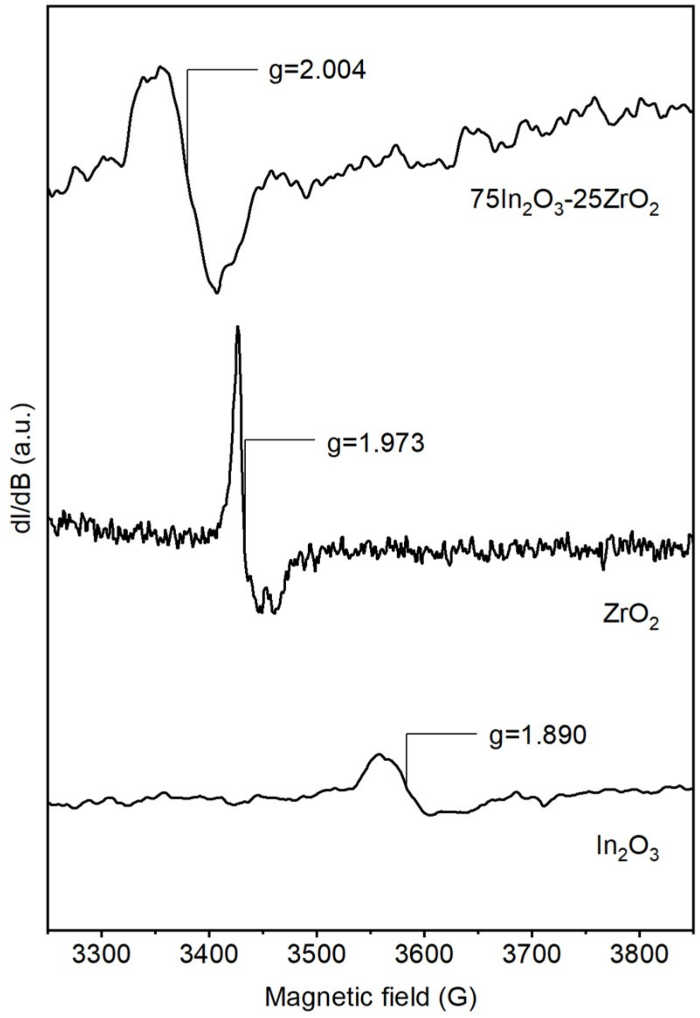

Figure S6 EPR spectra for $75In_2O_3$-$25ZrO_2$, In_2O_3 and ZrO_2.

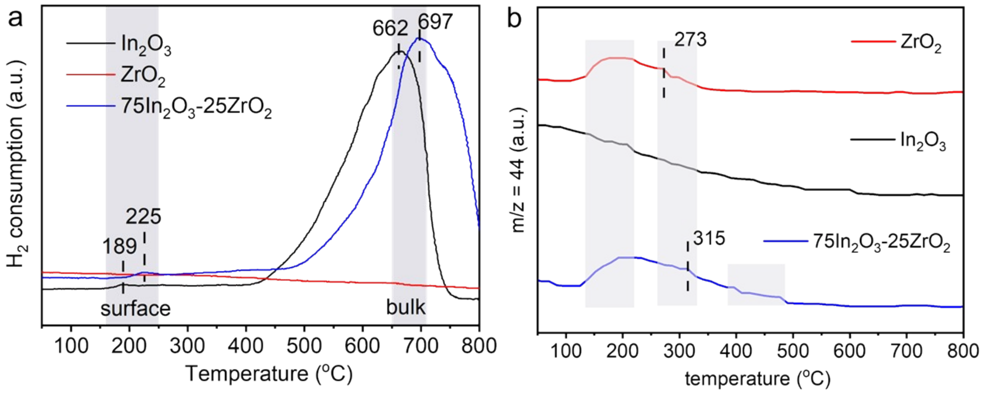

Fig. S7 (a) H_2-TPR profiles and (b) CO_2-TPD profiles of In_2O_3, ZrO_2 and $75In_2O_3$-$25ZrO_2$ catalyst.

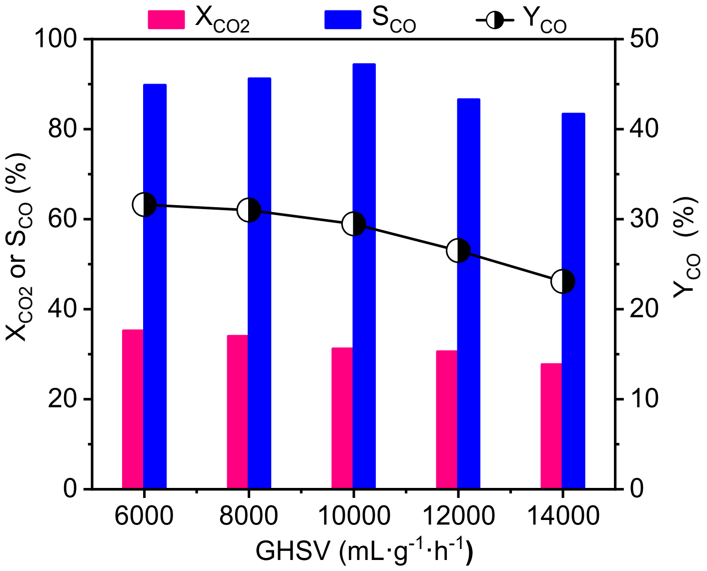

Figure S8 The influence of GHSV on the catalytic performance of $75In_2O_3$-$25ZrO_2$ (reaction conditions: 400 ^oC, H_2: CO_2: Ar ratio = 72: 24: 4, 0.1 MPa).

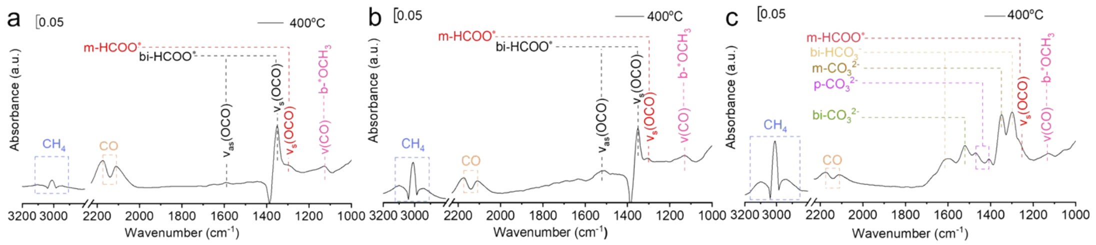

Figure S9 *In-situ* FTIR spectra of the reaction of CO_2 and H_2 over (a) 75In_2O_3-25ZrO_2, (b) In_2O_3, and (c) ZrO_2 (reaction conditions: 400 ^oC, 0.5MPa).

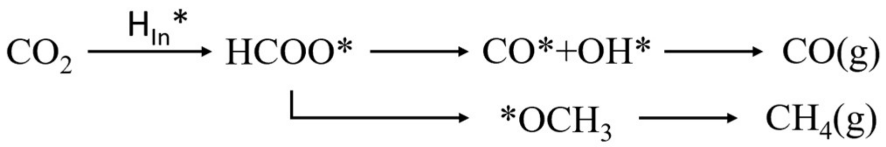

Scheme S1 Reaction mechanism catalyzed by $75In_2O_3$-$25ZrO_2$.

Reference

1 W. Wang, Y. Zhang, Z. Wang, J.-m. Yan, Q. Ge and C.-J. Liu, Catal. Today, 2016, **259**, 402-408.

2 M. Li, T.H.M. Pham, Y. Ko, K. Zhao, L. Zhong, W. Luo and A. Zutte, ACS Sustain. Chem. Eng., 2022, **10**, 1524-1535.

3 Z. Zhang, Y. Zang, F. Gao, J. Qu, J. Gu and X. Lin, New J. Chem., 2022, **46**, 22332-22340.

4 Q. Zhang, L. Pastor-Perez, Q. Wang and T.R. Reina, J. Energy Chem., 2022, **66**, 635-646.

5 L. Chen, D. Wu, C. Wang, M. Ji and Z. Wu, J. Environ. Chem. Eng., 2021, **9**, 105183.

6 Y. Liu and D.Z. Liu, Int. J. Hydrog. Energy, 1999, **24**, 351-354.

7 A.I.M. Rabee, D. Zhao, S. Cisneros, C.R. Kreyenschulte, V. Kondratenko, S. Bartling, C. Kubis, E.V. Kondratenko, A. Brueckner and J. Rabeah, Appl. Catal. B-Environ., 2023, **321**, 122083.

8 N. Ishito, K. Hara, K. Nakajima and A. Fukuoka, J. Energy Chem., 2016, **25**, 306-310.

9 S. Choi, B.-I. Sang, J. Hong, K.J. Yoon, J.-W. Son, J.-H. Lee, B.-K. Kim and H. Kim, Sci Rep, 2017, **7**, 41207.

10 W. Wang, Z. Qu, L. Song and Q. Fu, J. Energy Chem., 2020, **40**, 22-30.

11 S.S. Dang, B. Qin, Y. Yang, H. Wang, J. Cai, Y. Han, S.G. Li, P. Gao and Y.H. Sun, Sci. Adv., 2020, **6**, eaaz2060.

12 Y. Wei, F. Liu, J. Ma, C. Yang, X. Wang and J. Cao, Mol. Catal., 2022, **525**,

112354.

13 L. Lin, S. Yao, Z. Liu, F. Zhang, N. Li, D. Vovchok, A. Martínez-Arias, R. Castañeda, J. Lin, S.D. Senanayake, D. Su, D. Ma and J.A. Rodriguez, J. Phys. Chem. C, 2018, **122**, 12934-12943.

14 S. Collins, M. Baltanas and A. Bonivardi, J. Catal., 2004, **226**, 410-421.

15 F. Ouyang, J.N. Kondo, K. Maruya and K. Domen, Catal. Lett., 1998, **50**, 179-181.

16 S. Kattel, B. Yan, Y. Yang, J.G. Chen and P. Liu, J. Am. Chem. Soc., 2016, **138**, 12440-12450.

17 C. Yang, C. Pei, R. Luo, S. Liu, Y. Wang, Z. Wang, Z.-J. Zhao and J. Gong, J. Am. Chem. Soc., 2020, **142**, 19523-19531.

18 T.-y. Chen, C. Cao, T.-b. Chen, X. Ding, H. Huang, L. Shen, X. Cao, M. Zhu, J. Xu, J. Gao and Y.-F. Han, ACS Catal., 2019, **9**, 8785-8797.

19 W. Wang, Z. Qu, L. Song and Q. Fu, J. Catal., 2020, **382**, 129-140.

20 B. Liu, T. Fang and Y. He, Catal. Sci. Technol., 2022, **12**, 300-309.

21 K. Pokrovski, K.T. Jung and A.T. Bell, Langmuir, 2001, **17**, 4297-4303.

22 Y. Wang, L. Zhu, Y. Liu, E.I. Vovk, J. Lang, Z. Zhou, P. Gao, S. Li and Y. Yang, Appl. Surf. Sci., 2023, **631**, 157534.

23 L.Z. Gao and C.T. Au, J. Catal., 2000, **189**, 1-15.

24 G.C. Cabilla, A.L. Bonivardi and M.A. Baltanás, J. Catal., 2001, **201**, 213-220.

25 C.Y. Regalado Vera, N. Manavi, Z. Zhou, L.-C. Wang, W. Diao, S. Karakalos, B. Liu, K.J. Stowers, M. Zhou, H. Luo and D. Ding, Chem. Eng. J., 2021, **426**, 131767.
